# Understanding life together: A brief history of collaboration in biology

**DOI:** 10.1016/j.endeavour.2013.03.001

**Published:** 2013-09

**Authors:** Niki Vermeulen, John N. Parker, Bart Penders

**Affiliations:** 1 Centre for the History of Science, Technology and Medicine, University of Manchester, Simon Building, Brunswick Street, Manchester M13 9PL, UK; 2 Barrett, The Honors College, Arizona State University, P.O. Box 871612, Tempe, AZ 85287, USA; 3 Department of Health, Ethics & Society, School for Public Health and Primary Care (CAPHRI), Maastricht University, P.O. Box 616, 6200 MD Maastricht, The Netherlands

## Abstract

The history of science shows a shift from single-investigator ‘little science’ to increasingly large, expensive, multinational, interdisciplinary and interdependent ‘big science’. In physics and allied fields this shift has been well documented, but the rise of collaboration in the life sciences and its effect on scientific work and knowledge has received little attention. Research in biology exhibits different historical trajectories and organisation of collaboration in field and laboratory – differences still visible in contemporary collaborations such as the Census of Marine Life and the Human Genome Project. We employ these case studies as strategic exemplars, supplemented with existing research on collaboration in biology, to expose the different motives, organisational forms and social dynamics underpinning contemporary large-scale collaborations in biology and their relations to historical patterns of collaboration in the life sciences. We find the interaction between research subject, research approach as well as research organisation influencing collaboration patterns and the work of scientists.

## Introduction

In science, a single lifetime is often enough to witness major transformations.^1^ Though the 20th century witnessed major developments in physics research, its second half was marked by transformations in molecular biology. Nobel Prize winners James Watson and John Sulston both witnessed, contributed to, and chronicled these changes.^2^ Watson's ‘Double Helix’ recounts the reconstruction of the structure of Deoxyribose Nucleic Acid (DNA) in 1953, as published in a seminal *Nature* paper. He developed the model of DNA, together with Francis Crick, within the Cavendish Laboratory in the traditional English university town Cambridge. They worked relatively independently and the number of other scientists that figure in ‘The Double Helix’ is limited.^3^ Watson describes the scientific quest of a small group of scientists pursuing research in a small-scale academic environment. Sulston's story relays a completely different world. Though Sultson's career began in the worm research community in much the same small-scale academic environment as Watson – the Laboratory of Molecular Biology in Cambridge – his description of his later years deciphering the human genome illustrates a radically different world, involving the planning and adaptive management of a large, dynamic project with a clear mission, huge budget and expensive instruments involving hundreds of scientists in laboratories spanning the globe. Moreover, the exclusively academic environment is supplanted by an international and political setting, including academia, governments, funding bodies, business, media and the public.

As in molecular biology, so too has research in ecology undergone major transformations, transitioning rapidly from single-investigator studies conducted within a few square metres over a single study season to large, highly interdependent, transdisciplinary, cross-sectoral collaborations blending basic and applied science.^4^ Fred Grassle, a senior marine biologist collaborating in the decade long, international ‘Census of Marine Life’, witnessed and contributed to these changes. Grassle's interest in marine biology was triggered as an undergraduate when a biology teacher studying marine invertebrates invited him to study the mysteries of life at the sea bottom. He spent his early career at the Woods Hole Institute specialising in benthic ecology, and in 1989 founded the Institute of Marine and Coastal Sciences (IMCS) at Rutgers University. Believing that there was an insufficient focus on marine biodiversity, he also designed and initiated the Census of Marine Life–an ambitious, large-scale, international, interdisciplinary research project devoted to cataloguing all oceanic life. The Census has shown that the age of discovery is not yet over. It also created an international network of marine scientists, expanded the temporal range of marine research to include the past, present and future, and transformed research practice through the development of new technologies, databases, and new governance and communication strategies.^5^ Grassle was awarded several prizes as a result for his contributions to ocean science and an enduring place as a research pioneer witnessing and participating in major transformations in scientific practice.

These scientific biographies evince in personal terms broad and enduring cultural, organisational and historical shifts in the ways in which biologists collaborate and relate to their study objects. This article focuses on these transformations in the orchestration, conduct and structure of contemporary collaborations in the life sciences. We consider factors related to the rise of large, complex, interdependent collaborations in the life sciences and how these contributed to the changes in ‘doing biology’ that Grassle, Watson and Sulston and their contemporaries witnessed over the course of a few decades. We do so by reviewing evidence of rising rates of collaboration in the life sciences while also showing that collaboration has been common throughout their history. On the basis of this historical overview we discuss differences in the developmental trajectories of collaboration in molecular biology and ecology, arguing that ancestral epistemological and organisational legacies continue to structure and inform contemporary research practice. Doing so provides a general understanding of the causes and consequences of changing patterns of collaboration in biology while specifying and analysing important differences in lab- and field-based research. This distinction is one of degree – research blending elements of lab and field biology have always existed – but different environments impart important consequences for the ways in which science is performed and the kinds of outcomes that are created. We conclude by reflecting on the overlap between field and lab research and the potential courses life science collaborations may take into the near future.

## The growth of biology

Scientific collaboration is on the rise. Examinations of the 2.4 million scientific articles produced by the top 110 US universities between 1981 and 1999 reveals that research team size increased by 50% during this period. This trend accelerates over time from a 2.19% annual rate of growth in the 1980s to a 2.57% rate in the 1990s (an acceleration factor of 17%). Average distance between collaborators also increased, with the annual rate of growth of average miles between collaborators within US universities rising from 3.53% in the 1980s to 4.45% in the 1990s. During this same period rates of collaboration between US and foreign universities increased five-fold.^6^ Similarly, analyses of 19.9 million articles collected by *Web of Science* (1955–2000) indicate that team size increased in 99.4% of science and engineering subfields.^7^ Clearly, scientific collaborations are getting bigger and more international.

Collaboration in biology follows the same patterns. Considering all articles in the *Web of Science* database, the size of research teams in biology more than doubled from 1955 to 1990 – a trend slightly higher among molecular biologists (increasing 129%) and slightly lower among ecologists (increasing 83%).^8^ Among the top 110 US universities average research team size in biology increased 52% from 1981 to 1990. With the single exception of medicine, biological collaborations also experienced the greatest growth in average distance between collaborators.^9^ Within the European Union, during the period 1998–2003, the life sciences became the most collaborative field after physical, chemical and earth sciences.^10^ Collaborations in the life sciences are most often intra-EU collaborations, but they also rank as the second field of extra-EU collaborations.^11^

Quantitative studies clearly indicate a rise in collaboration, but leave unexplored the reasons for this increase and the precise character of the collaborations, begging many questions. One study suggests that the acceleration of collaboration has been made possible by a sharp decline of the costs of collaboration,^12^ but is that the only reason, or might the character of scientific questions, their subject matter or the technologies employed also be of influence? Moreover, is the increase driven by purely scientific motives, or do societal developments such as changing demographics increase the interest in human life and health, while issues such as climate change and biodiversity increase interest in non-human life? What can the tendency to collaborate within the European Union tell us? Are we witnessing cultural proximity at work, or can the preference for intra-European collaboration be explained by patterns of research funding? And are collaborations in the life sciences one big category, or can we also find differences within biology when looking into its sub-disciplines?

In short, collaboration in the life sciences is increasing – but *why*? As the social scientist Edward Hackett points out, ‘These deceptively simple questions have elicited and qualified answers.’^13^ Here we engage in such elicitations and qualifications to better characterise the changing nature of collaboration in the life sciences, beginning with a discussion of various definitions of collaboration and following with a historical accounting of its origins and growth, which is richer and more nuanced than can be garnered from quantitative approaches.

## Studying collaboration

Science studies scholars have adopted varying definitions and approaches for conceptualising and researching scientific collaboration. The notion of ‘co-laboring’ can be seen as the literal roots of collaboration. Definitions of scientific collaboration vary from broad definitions involving co-working to more delimited definitions requiring teamwork with shared goals, ‘such as formulating or testing particular empirical hypotheses’, and shared products, ‘such as co-authored papers.’^14^ Similarly, scientific collaborations can also involve non-scientists or extend beyond scientific goals.^15^ Moreover, scientific collaboration can be informally organised or firmly institutionalised as in ‘an institution for conducting ‘big’ science, work that involves coordinating many people and substantial resources for long periods of time’.^16^ Hackett's definition – the one adopted here – provides a productive middle-ground blending a broad scope with an explicit focus both on the cooperative nature of the enterprise and the types and nature of the resources they form to exchange: ‘collaboration is a family of purposeful working relationship between two or more people, groups, or organisations. Collaborations form to share expertise, credibility, material and technical resources, symbolic and social capital’.^17^

Most of the studies that define and describe scientific collaboration are based on investigations of physics or space research. They study for instance an organisation like CERN in Geneva, where large-scale instruments are built to detect the very substance of matter: sub-atomic particles. As these so-called detectors are very big, single institutes or nations are unable to afford their construction, requiring collaboration. Similarly, space research concentrates around large-scale technology, and requires a centralised, hierarchical and tightly integrated organisational structure for successful execution. While a large body of research in science studies has demonstrated the centrality of systems and technologies for the organisation of collaboration, a concise and coherent narrative on collaboration in the life sciences remains absent. Nevertheless, the existing literature offers valuable insights. While acknowledging the complexity of the phenomenon and noting the relative lack of qualitative studies, they advance various approaches for collaborating, reasons for doing so, as well as considering the structures in which collaborations occur.

Reasons for scientific collaboration vary. The development of large, fabulously expensive instruments is a reason to share costs and collaborate. Other motives include the need for complementary specialties or disciplines, as well as pressure for societal relevance, decreasing travel and communication costs, and to increase scientific credibility at the level of the project or even discipline. Research has also demonstrated the importance of strong interpersonal relationships and deep emotional commitments to the group and its ideas for motivating and structuring collective scientific work. Additionally, collaboration can be stimulated by funding incentives, political motivations, or simply because it is viewed as good in and of itself. Overall, collaboration is driven by a variety of purposes and reasons, at least some of which are ubiquitous across disciplines.

Structures of scientific collaboration also vary. Shrum et al. (2007) distinguish four different ways to structure collaboration on the basis of their mix-methods study of 53 multi-institutional collaborations in particle physics, space sciences and allied disciplines.^18^ *Bureaucratic collaborations* have a hierarchical authority structure, written rules and regulations, formalised responsibilities, and a specialised division of labour, while *leaderless collaborations* are similarly formal but do not have a designated scientific leader and lack hierarchical management. In contrast, *non-specialised collaborations* have designated scientific leaders and are hierarchically managed, but are less formalised and differentiated than bureaucratic collaborations. Finally, *participatory collaborations* are highly egalitarian, with participatory and consensual decision-making, no formal organisational structure, and limited regulatory powers among scientific leaders. This last type is typical of particle physics, while the other types were found to exist across the investigated disciplines. However, which structures are present in biology?

Some answers on this question may be found in existing research. Sociologist of science Knorr-Cetina compared collaborative practices in high-energy physics and molecular biology during the 1980s, concluding that in opposition to the large transnational collaborations of physics, biology is an individual centred, non-collaborative science.^19^ However, in the 1990s several studies on the Human Genome Project began foregrounding collaboration in biology. The Human Genome Project was often viewed as the first true large-scale collaboration in biology, giving rise to a variety of publications discussing issues related to collaboration such as structure, data exchange and public-private competition.^20^ Peter Glasner (1996) used the term ‘co-laboratory’ to indicate that the project was built of different international laboratories working together to produce the human genome sequence. The Human Genome Project also gave rise to debates about the benefits and demerits of collaboration, including arguments that large-scale projects can industrialise, bureaucratise and politicise research, potentially diluting scientific autonomy, creativity and job satisfaction. To illustrate, genome sequencing was portrayed as “massive, goal-driven and mind-numbingly dull”.^21^ These debates reflect more general critical approaches to collaboration, which can be difficult, time consuming, and impose substantial coordination and communication costs.^22^

Overall, studies of collaboration in the life sciences are severely lacking, and the scholarship that does exist has focused primarily on the Human Genome Project, leaving other areas of biology unexplored. It is unclear how variable patterns of collaborations are in the life sciences, and if biologists collaborate for the same reasons and in the same ways as scientists in other fields.

Given these substantial uncertainties, the rising prominence of the life sciences, their increasing societal relevancy and the degree of societal investment in them, this situation demands amendment. In the following we consider how and why biologists collaborate, and why they are doing so with greater frequency and in collaborations of greater scope, expense, complexity and intellectual ambition. Furthermore, we move beyond the current emphasis on molecular biological research taking place in laboratories, paying equal shrift to the organisation and changing patterns of collaboration in ecology and the field. We sketch the differences between field-based and lab-based data collection and analysis in the life sciences. Apropos to this endeavour, we begin with an historical overview of collaboration in these different research settings.

## Exploring historical roots of collaboration in biology

Although collaborative approaches to knowledge production are becoming more commonplace, their roots can be traced back centuries. Collaboration in biology is not new. Historical precursors exist in both natural history and laboratory biology, creating enduring epistemological and organisational legacies.

### Collecting life collectively: collaboration in field biology

Historically, the most important reason for cooperation in field biology was the dispersed character of biological material. No one person can possibly get an overview of the variety of life on Earth, and so it makes sense that natural historians were part of the first forms of scientific collaboration. Described as the ‘grand alliance’ between science and exploration in the 17th century, they joined expeditions exploring the unknown world in order to describe, collect and catalogue new species, accumulating facts about plants and animals that were brought together in private collections or natural history museums. While in 1600 CE only around 6000 plant species of plants were known; by 1700 CE botanists had collectively discovered 12,000 new species, with similar accumulations in zoology. This advanced classificatory schemes – leading to Linnaeus's *Systema Naturae* (1735) and the evolutionary theories of Lamarck (1809/1984) and Darwin (1859)^23^ – but also significantly changed the ways in which biologists related and communicated with one another and acquired their research materials. Naturalists did not only join forces with world explorers, they also set up networks of scientific assistants and colleagues. Linnaeus, for instance, often used his (former) students to find new specimens in different parts of the world to bring back to his botanic garden in Uppsala.^24^ Additionally, infrastructural developments in transportation and communication technologies were crucial for these first, loosely structured forms of collaboration.

Early scientific expeditions gradually evolved into more coordinated multi-disciplinary research programmes, initially taking the form of scientific agencies, thematic years or decades. An early example in the United States was the establishment of scientific agriculture through the Morrill Act, while international example include Baird's Commission on Fish and Fisheries (1871) orchestrating international research on fish and fisheries and the International Polar Years (1882–1883/1932–1933) concentrating international research efforts to investigate North and South Pole: ‘The experience gained by scientists and governments in international cooperation set the stage for other international scientific collaboration’.^25^ The success of the polar years led to the organisation of the International Geophysical Year (1957–58), which in turn functioned as a model for the International Biological Programme (1968–1974), advanced by the International Union of Biological Sciences, receiving important support from the US government. The programme investigated ‘The Biological Basis of Productivity and Human Welfare’ and “caused many nations to learn how to work together in scientific research with the highly practical purpose of improving the life of humankind”.^26^

The IBP can be seen as the first time in which ecology became big science.^27^ Although according to the original conception genetics and human population studies would be part of the core of the programme, in the light of developments in large-scale physics and emerging approaches to cybernetics, upcoming environmental concerns and systems ecology with its promise to control nature became the central issues of the programme. In the same period, physicists at Oak Ridge and Brookhaven national laboratories interested in the effects of radiation started to work together with ecologists to conduct large-scale experiments introducing radio-isotopes into local environments to trace energetic and material flows through ecosystems.^28^ These experiments forwarded systemic approaches to ecosystem studies and increased the legitimacy of ecological research in the eyes of funding agencies, the public and other disciplines. They also left a lasting legacy in the form of region-specific cancer clusters.

The Long-term Ecological Research Network was another major development in big field biology. Created to enhance understanding of deep-time ecosystem evolution, an initial set of six research sites was created in 1980, to be studied and funded in perpetuity and since then many more (inter)national sites have been constructed. The network has increased collaboration between site members, added new disciplinary dimensions to ecosystem science, and enhanced understanding of long-term ecosystem dynamics. It also inspired the new National Ecological Observatory Network, and project promising to automate field data collection through the construction of a network of observational platforms containing instruments and sensors capable of remotely measuring and communicating field data. The implications of these new technologies for collaboration in field biology are immense, allowing access to otherwise inaccessible ecosystems and as yet undreamed of forms of collaboration.

### Dissecting life together: collaboration in laboratory biology

Today's dominant image of biology is of a bench science confined to a laboratory. The natural history model of exploratory research gave way to the age of analysis and experimentalism, bringing a more mechanistic view of life with attendant efforts to control nature and create novelty within the lab.^29^ These developments have to be placed against the background of social transformations, such as the growing importance of the nation state, institutionalisation, professionalisation and industrialisation. Science became the motor of the state, and the creation of the lab was flanked by discipline formation and the emergence of the ‘research’ university. Nascent forms of collaboration were present during this era, including research schools, interdisciplinary collaboration and early cooperation between science, industry and government.

The Cambridge school of physiology exemplifies collaboration in late Victorian England.^30^ Led by Michael Foster, the school had a profound influence on the development of physiology, being part of a broader trend towards laboratory-based research schools and collaborations in which scientists pursued coherent research programmes, engaged in direct, continuous social and intellectual interaction, and transmitted craft skills of investigation between colleagues and from master to pupil. Another early form of research coordination can be found in the US, where during the 1930s the Rockefeller Foundation stimulated interdisciplinary investigations into the organisation of life through its ‘Science and Man’ programme.^31^ These efforts blended biology, chemistry and physics with a specific focus on genetics, contributing to the emergence of a new biology.

The emergence of large biomedical complexes focusing on medicine and involving industrial collaborators also exemplifies an early form of collaboration in laboratory biology. First forms of larger-scale physics research emerged in the first decades of the twentieth century and gave rise to biomedical research such as radiobiology. Also collaborations with industry began in the 1920s and 1930s when American pharmaceutical firms invested in research as a competitive strategy in medical reform movements aiming to make science the basis of therapeutic practice. Firms opened in-house laboratories and funded academic researchers, stipulating that new processes and inventions be available to the firm. These collaborations set the stage for large-scale arrangements during World War II: “‘Big’ biology, performed in comparatively large groups with substantial budgets, was already commonplace by 1941, financed by drug and chemical companies or, in selected fields less favoured by industry, by philanthropies such as the Rockefeller Foundation”.^32^ In World War II large-scale US research projects focussed on producing medicine, for instance developing penicillin and blood products.

Collaboration in laboratory biology is usually depicted as developing in interaction with investigations into genetics. Watson and Crick shared the Nobel Prize for their DNA work, leading to the development of molecular biology in the late 1950s and 1960s. The European Molecular Biology Organisation (EMBO) was created in 1964, followed by the European Molecular Biology Laboratory (EMBL) in 1974. Next to this international institutionalisation that mimics the centralisation of research in particle physics, more decentralised international mapping and sequencing networks focused on model organisms *Drosophila* and *C. Elegans*. Scientists studying fly and worm began exchanging research materials, divided labour across laboratories and stimulated the development of gene mapping and sequencing technologies. These stand as important forerunners of the Human Genome Project, structuring future efforts to dissect the essential components of life and disease, helping to ensure that “Like it or not, big biology is here to stay”.^33^

## Contemporary patterns of collaboration in lab and field biology

As indicated above, collaboration in biology has increased substantially and takes different shapes in field and laboratory work. While collaboration in field biology was motivated by the dispersion of research materials, collaboration in laboratory biology has only recently grown in size and complexity with the advance of molecular, genomic and post-genomic research. In contemporary field biology collaborations range from exploratory expeditions to large-scale, long-term, ecosystem assessments with highly technical observational platforms. Being faithful to the context of exploration, the collaborations consist of decentralised, individual components or field-sites that collect species in (virtual) databases to map and model relationships in order to describe and understand life on earth and its development, albeit in increasingly technically sophisticated ways. In contrast, laboratory biology takes nature inside its walls to analyse and experiment, while emerging in a period when science became institutionalised and part of national governance structures. In line with these historical developments, laboratory-based collaborations are also determined in part by institutional and (inter)national integration in networks. In interaction with technological developments, laboratories become increasingly connected and these so called “collaboratories” are increasing in size, diversity, complexity and scope.

Based on these differences, the question arises: How do the social, technical and intellectual inertia imparted by these two developmental strands influence collaborations in field and laboratory biology today? Contemporary patterns of collaboration are determined both by motivations to collaborate and the various methods for doing so, the latter constituting working and governance structures of collaboration. Reasons for collaboration in biology differ from other fields, and between subfields within the life sciences. The result is that the rationale for Grassle to start the Census of Marine Life may be remarkably different from Watson's and Sulston's reasons for enlisting in the Human Genome Project. Similarly, structures of collaborations vary. While collaborations in particle physics or space research are centrally organised around large instruments, collaborations in biology have a more networked structure. However, field and laboratory biology show differences beyond distinct reasons to collaborate, including the ways in which connections get shaped, the governance of collaboration and the goals of collaboration. The following overview of contemporary collaborations in biology, including their motivations and structures, uses the Census of Marine Life and the Human Genome Project as exemplary case studies for highlighting these differences, but is additionally rooted in detailed research into twelve recent collaborations in lab and field biology (six field-based and six lab-based) and literature on the Human Genome Project.^34^ We close by reflecting upon and tracing the historical continuities and legacies that can be observed in contemporary collaborations.

### Reasons for collaboration in field and laboratory biology

In field biology the geographic dispersion of research materials continues to be one of the most important reasons to collaborate.^35^ For instance, the Census of Marine Life was instantiated because though the oceans cover seventy percent of the earth's surface area its inhabitants remain an enduring enigma. Moreover, marine biological research was scattered around the world with no systematic, comprehensive global initiative to describe and catalogue marine life. For this reason Grassle and colleague Jesse Ausubel began plans to catalogue all marine life, spawning the contemporary global census of marine life – a project involving fourteen different field projects, scores of scientists, and geographic coverage ranging from the coastal waters to the vast and mysterious benthic depths. Standardisation of data and findings has also always been an important impetus for collaboration in biology. Though also motivated by a range of other factors, collaborations within the Long-term Ecological Research Network and the National Ecological Observatory Network were created in large part to gather, collate and analyse data of broader spatial and deeper temporal scope than had been previously possible. These large datasets are often then used to develop computing and models for discovering new patterns and generating new hypothesis to improve our understanding of life and its dynamics.

Some have claimed that a lack of large-scale instruments has limited collaboration in laboratory biology, but it is nonetheless the case that molecular biological instrumentation has become bigger, more complex and substantially more expensive, resulting in the concentration and centralisation of research efforts. For instance, the development of genome sequencers was a fundamental aspect of the Human Genome Project, their development made sequencing the entire human genome feasible, while simultaneously giving rise to newer and faster sequencing technologies. Interestingly, the development of sequencing technologies was not fundamentally motivated by a desire to centralise and enlarge collaboration in laboratory biology, but rather to increase the speed with which samples could be processed and data produced and analysed. The desire to increase the velocity of scientific analysis is also apparent in nutrition science, where large collaborative initiatives aim to forward molecular analysis over time-consuming physiological evaluations. In both cases time compression through advanced instrumentation and internationalisation of research dovetails with a third motivation for collaboration: standardisation. Common standards are requisite for making compatible data from multiple sources and locations. For instance, the universal coding of DNA through the letters A, G, C and T and the negotiation of common research methods to decipher the code was essential to the success of the HGP. Similarly, the establishment of a standard platform for large-scale measuring of gene protein expression and metabolite concentration in molecular nutrition science is both a reason to collaborate in European nutrition science, and the main cause for its enduring success.

Across the range of case studies, reasons to collaborate vary, most common being instrument access, geographical and temporal compression and standardisation. These reasons are present in laboratory and field biology alike, but differ in terms of relative degree between these subfields. As in other disciplines, standardisation of instruments, objects of study and data collection protocols is also an important reason to collaborate in biology. Alternately, temporality and geographic dispersion provide greater incitation to collaborate in biology relative to other fields.

Several answers may be given to the question of why biology is experiencing such a meteoric rise in the extent and intensity of collaboration. Collaboration in physics and space science were primarily driven by national concerns for military defence and the search for cheap and enduing energy supplies. The same is not the case for collaboration in biology, which can rather be seen as arising out of social concerns related to human health and well-being. This is in part reflected in a doubling of the budget of the US National Institutes of Health and similar increases in EU member state's funding of genomic and post-genomic research. At the same time, increasing environmental pollution, climate change and loss of biodiversity have increased attention to the study of non-human life, albeit at much more modest funding levels. In sum, these societal factors, together with an increasing political emphasis on the relevance and application of research, have combined to increase markedly the magnitude and complexity of collaboration in biology.

### Structures of collaboration in field and laboratory biology

The ‘structure’ of a scientific collaboration refers both to the pattern of relationships among scientists, technicians, support staff and other actors, instruments and study objects as well as the governance structure designed to oversee and regulate it. Particle physics and space research collaborations tend to be organised around large instruments. In biology, collaborations tend towards a less centralised, more networked pattern of relationships due to the dispersion of research materials in field biology, and the informational turn in laboratory biology, allowing the construction of databases which do not require researchers to be physically co-present. At the same time, large-scale collaborations in biology do show central forms of coordination. Both the CoML and the HGP combine a central governance structure with globally dispersed research locations. However, as these cases stem from different research settings, they also allow us to show how different ancestral epistemological and organisational legacies shape contemporary collaborations.

Importantly, form tends to follow function – the character of the research determines the way in which the research collaboration gets organised. So motives to collaborate and research objectives play an important role in the structure of the collaboration that materialises. Additionally, both the organisational form and the governance structure are to a large extent determined by the type of funding available and the specific accountabilities attached to the funding source. The Census of Marine Life and the Human Genome Project have different motivations, objectives and funding regimes, and this results in different levels of integration and distinct forms of leadership.

The Census of Marine Life has been initiated to ‘assess and explain the diversity, distribution, and abundance of marine life in the oceans–past present and future’. This objective was translated into the building of a database with records of current species, as well as historic research and modelling to map temporal transformations. The bulk of scientific work within CoML consisted of seventeen field projects detecting species to enter in the database. Ideally, records were configured according to database specifications, but as all available information on life in the oceans is valuable for the objective of CoML, the database is also open for non-standardised contributions and for marine biologists outside CoML. As funding for such a large-scale project was unavailable, the Sloan Foundation provided funding for global governance and project oversight, while the actual research was funded by national and regional research funding programmes. Consequently, CoML consisted of a patchwork of local research projects with their own management structures governed by an international Scientific Steering Committee. As a result, CoML had a very decentralised character and the leadership needed to balance guidance of a global scientific community of marine biologists while accommodating participation by small-scale research projects with different locally determined requirements. These smaller projects did not depend on each other in their work, so the structure of CoML could be less hierarchical and more democratic, having open boundaries in order to gather as much data as possible.

In contrast, collaboration in the HGP was mainly motivated through the wish to speed-up the mapping of the human genome by compressing research time through teamwork. While the Human Genome Project objective was also database construction, contributions were strictly regulated. Starting with a selection of participating labs, work was divided, granting each lab the responsibility to map a specific chromosome. Moreover, standardisation had to take place in order to make the research results comparable and compatible. Thus, interdependence between research teams was much higher, requiring a more hierarchical decision structure. As funding for both research and management came from various national funding bodies and the Wellcome Trust in the UK, governance became concentrated in national components led by eminent scientists such as Watson (US) and Sulston (UK), who also took care of international coordination. As a result, the HGP was a more finely structured and more hierarchically organised research network. Furthermore, collaborative boundaries were closed as the emphasis was on efficiency and only trusted partners that assured quick, credible and compatible research results were incorporated.

Finally, the integration of research results in society has led to differences in the governance structures of the Census of Marine Life and the Human Genome Project. After all, scientific practice and the social organisation of science intertwine to shape scientific results that impact policy and the whole of society. In CoML, the objective was the mapping and modelling of marine life, making (unknown) life visible for both scientists and the public. This required, in addition to scientific publications, newspaper articles, coffee table books, exhibitions and a movie entitled ‘Oceans’, which screened in cinemas worldwide, showing the wonders of ocean life and raising awareness for protecting marine life. CoML demonstrated a 50% reduction in the diversity of fish in the open ocean coinciding with the emergence of large-scale commercial fishing; a fact that resulted in global news coverage and policy discussions. While the public was not forgotten in the HGP, emphasis was more on applications and implications of research and private-sector relations. The HGP aimed to improve knowledge of human life, as well as to find genetic bases of disease to find cures. Anticipating important social implications, the HGP studied ethical, legal and social implications (ELSI) of its work in parallel. Its application-orientated nature also raised interest from private companies, expecting opportunities for profit. More concretely, this led to the competition with Craig Venter's Celera, the private company that also sequenced the human genome. While the HGP has not provided the key to cure all diseases, it has resulted in important progress in health care.

The comparison between the structures and governance programmes of the Census of Marine Life and the Human Genome Project exemplifies differences similarly prominent in our other ten case studies. Where laboratory work is more strictly governed and oriented to practical applications and innovations, field collaborations tend to be more democratic and more geared to description and demonstration. For instance, although both the Long Term Ecological Research Network (LTER) and CoML gather different sorts of data in different types of ecosystem, both networks are characterised by a decentralised governance structure and organic growth. Indeed, even within the different LTER sites research work is performed and reported on independently and is only connected through so-called ‘synthesis themes’. In both cases research is characterised by high autonomy, low task interdependences, and high task uncertainty.

Alternately, and similarly to the HGP, large-scale genomics and post-genomics networks that followed the HGP are more centrally and tightly directed. A good example is the development of virtual models of (parts of) life that is the objective of collaboration in systems biology. In the case of the European Systems Biology of Microorganisms (SysMO) consortium,^36^ the modelling of a yeast cell for instance not only requires genes to be mapped, but also the mapping and integration of all other cellular components. It is imperative to perform research into the diverse components of the cell with the same standards in order to be compatible in a digital model that mimics the processes in a living cell. The relationship between low autonomy for researchers and high interdependency of research tasks is confirmed when turning to nutritional research collaborations, such as the European Nutrigenomics Organisation (NuGO) and the public–private Top Institute for Food and Nutrition (TiFN), both of which are exploring on a molecular level the effects of food on health in the laboratory. Also, within applied molecular research, such as the VIRGO consortium that uses genomics technologies to improve insight into diagnostics and therapy of virus infections, typically requires greater integration of research activities (e.g. as in the pharmaceutical industry which works with pipelines of research in which all the subsequent research steps are dependent on each other). In each case research is characterised by high task interdependence, relatively high task certainty, and low individual autonomy.

Although the distinction between field and laboratory biology clearly influences the structure and governance of collaboration, is has to be noted that the border between field and laboratory biology is not always clear-cut. In historical and contemporary biology collaborations exist that cannot be classified as either field or lab biology. For instance, at the beginning of the 20th Century a tense relationship developed between these two approaches, leading to hybrid forms of research.^37^ While originally laboratory biology brought the field into the lab – standardising nature and making it possible to analyse and experiment in a controlled environment – laboratory work was soon preferred and considered the ‘proper’ way to study life. As a result, field researchers “felt bound to use lab methods and understood that their own practices and achievements would be judged by lab standards. …All lived to some degree in the shadow of laboratory science, and their successors still do.”^38^

More recently, a counter-movement has emerged recognising the value of conducting experiments in the field, creating hybrid approaches to biological research blending the logic, control and vocabulary of experimentalism with the natural environments, unexpected influences and potential serendipity of field work. Examples include ‘exploratories’ to understand biodiversity and its role in ecosystem processes and land use management in Germany, and the National Ecological Observatory Network (NEON) to conduct real-time ecological studies spanning all levels of biological organisation and temporal and geographical scales in the United States.^39^ These collaborations consist of distributed arrays of highly technical environmental sensors producing huge amounts of data requiring substantial standardisation and synthesis to produce meaningful results. Within these associations experimental and field biology combine into new hybrid forms defying easy categorisation.

## Collaboration in lab and field biology: reflections and predictions

The study of scientific collaboration is still a budding field, and only collaboration in physics is well documented. Collaboration in biology presents perhaps an even more interesting subject of study, ranging from its historical roots in natural history expeditions to current large-scale efforts in ecology and molecular biology. Teamwork in biology has increased substantially through a combination of increasing research funding, greater attention to research into life and transformations in the ways in which biologists position their research. CoML and HGP are exemplars of collaboration in field- and lab-based contemporary biology, exposing different characteristics, structures and dynamics of collaboration, each mirrored in data from our other case studies, internationally spanning field and laboratory. As a result, this brief excursion into past and present of collaboration in biology indicates that the organisation of science can be neither detached from its content nor from its past. Collaborative differences between laboratory and field based research can be traced to their subject matter and research approach, but the shape and internal dynamics of collaborations should also be understood against the backdrop of the historical legacies drawn from preceding collaborations and collaborative styles of work.

The history of collaboration in biology shows how developments towards collaboration in field and laboratory biology emerged in different periods, each with its distinct organisational character. Collaborations in field biology continue traditions in natural history by collecting and mapping the diversity of life on earth and in the oceans. A collective effort form the start, such collaborations are characterised by dispersed field sites and low interdependency between different research tasks, giving scientists involved ample autonomy in performing their research. In contrast, in lab biology where co-authorship and teamwork only recently increased with the focus on molecules as the basis of life, collaborating scientists experience less autonomy as research has higher levels of interdependency and an enhanced focus on applications. Possibly, the different timeframe in which lab-based biology grew large – a time of nationalisation, professionalisation and industrialisation – has influenced the formalisation and applied character of these collaborations. In (post-)genomics research understanding is geared towards innovation, which requires higher levels of integration, while ecology research is primarily oriented towards the understanding nature and environmental change, allowing more decoupled forms of organisation. This different orientation of molecular biology and ecology also causes a difference in financial resources for collaboration, as the goal of improving human health attracts more research funding than increased understanding of basic environmental processes.

Observing the increase of collaboration in the life sciences also results in appreciation of the amount of labour invested in issues of alignment, organisation and communication – key components of the production of scientific knowledge taking place inside collaborations.^40^ Collaborations also have considerable effects on scientific work life and career development as biologists are increasingly required to balance and mediate between the roles of researcher and manager. Biographies are valuable sources of information for understanding such issues, as we have shown by interweaving the stories of Sulston, Watson and Grassle throughout this text. It is within biographies that lived experiences of collaboration are experienced and felt. “I had not learned to be a manager”, Sulston writes, clearly identifying the changes he witnessed in the world of molecular biology, “[but this] was going to lead to a big management structure”.^41^ Even more strikingly, he experiences the changes to his career as a coming of age – not just of himself, but of the community of biologists at large: “As biologists we had lost our innocence. We were out in so-called the real world”.^42^ In fact, researchers have actively stimulated the growth of networks, expanding the scale of their efforts both globally and over time across the life sciences. Scientists such as Grassle, Watson and Sulston have been actively involved in the transformation towards more and bigger collaborations, thereby aiding biology in claiming a bigger role amongst the natural sciences.

It will be interesting to see how biology's biographies further develop. Although the main characters in this paper are still with us, they have retired and yielded control to the next generation. What will the future of biology look like? How will transformations in ecological research transform our visions of life and what will developments in molecular and systems biology bring in the upcoming decades? Projecting the (recent) historical developments into the near future, we expect collaboration in the life sciences to continue the current trend of blending analysis in both field and laboratory, creating hybrid knowledge production systems with an emphasis on the integration of knowledge. Blending is already visible in recent developments in ecology, for instance in projects which bring the lab into the field. Research traditions in ecology and molecular biology are also merging in a quest for a unified understanding of interactions between evolution, development and ecosystems. Moreover, scientists brought together by the US National Center for Ecological Analysis and Synthesis analyse and synthesise data from field sites around the world to examine broad-scale, long-term ecosystem dynamics, effectively removing the ‘field’ from field biology. This integration of data in ecology mirrors current developments in lab biology where we find an increase in systems approaches to model (part of) organisms, and in both research areas data integration requires further advancement.^43^ Increased pressure for societally relevant results is also likely to further tighten the relationship between science, politics and industry. Both the process of blending and of greater cross-sectoral pressures will increasingly take place on a global scale, recognising the global dimension of health and environmental problems. This enhanced geographic scale will allow for large instrumentation platforms and massively interlinked, yet distributed data infrastructures. Interestingly, this might also mean a shift in the international centre of gravity for knowledge production: out of North-America and Europe, into the rest of the world (e.g. Asia). Collaboration will continue to be one of the driving forces that change the face of science and young scientists and research managers will experience shifts during their career as dramatic as Watson, Sulston and Grassle did during theirs.

## Footnotes

1 The authors are grateful for the support of CSG Centre for Society and the Life Sciences at Radboud University Nijmegen, the Netherlands, providing us with the opportunity to further integrate our individual work on collaboration in the life sciences. Additionally, we thank the Department for the Social Studies of Science of the University of Vienna for their hospitality and the Wellcome Trust for support.

2 Watson, J. D. (1968). *The double helix: a personal account of the discovery of the structure of DNA*. New York: Atheneum; Sulston, J. and Ferry, G. (2002). *The Common Thread: A Story of Science, Politics, Ethics and the Human Genome*. London: Bantam Press.

3 Jim and Francis are accompanied by Maurice Wilkins and Rosalind Franklin, with occasional interactions with Max Perutz, John Kendraw, and Sir Lawrence Bragg. Those beyond this small group are of secondary influence. To many, it is a recognisable narrative of ‘little science’: the story of bold scientific heroes gaining ground on the unknown. This caricature of science is annually reinforced through the well-known Nobel Prize and its laureates. The individual character has led to the ‘HGP Nobel Prize problem’ – a concrete problem highlighting the mismatch between the collectivity of scientific practice and the individualisation of its assessment. See Zwart, H.A.E. (2010). The Nobel Prize as a Reward Mechanism in the Genomics Era: Anonymous Researchers, Visible Managers and the Ethics of Excellence. Bioethical Inquiry 7: 299–312.

4 We base the arguments in this paper largely on our earlier work on collaboration in biology in which we also refer substantially to literature on collaboration and related issues that has informed our work. When we do not give specific references to other sources here, the relevant references can be found in earlier papers: Parker, J. (2006). *Organisational Collaborations and Scientific Integration: The Case of Ecology and the Social Sciences*. PhD Thesis, Arizona State University; Parker, J.N, Vermeulen, N. & B. Penders. (2010). *Collaboration in the New Life Sciences*. Farnham: Ashgate; Parker, J.N. & E. Hackett (2012). “Hot Spots and Hot Moments in Scientific Collaborations and Social Movements.” *American Sociological Review*, 77(1): 21–44; Penders, B. (2010). *The Diversification of Health. Politics of large-scale cooperation in nutrition science*. Bielefeld (D): Transcript Verlag; Penders, B., Horstman, K. & Vos, R. (2008). “Walking the line between lab and computation: the ‘moist’ zone”. *BioScience*, 58 (8): 747–755; Vermeulen, N. (2009). *Supersizing Science. On Building Large-scale Research Projects in Biology*. Maastricht: University Press Maastricht; Vermeulen, N., Parker, J.N. & B. Penders. (2010). “Big, Small or Mezzo?: Lessons from Science Studies for the ongoing debate about ‘Big’ versus ‘Little’ Science”. *EMBO Reports*, 11, 420–423; Spruit, S., Schuurbiers, D. & Penders, B. (2012). *Embedding Nutrigenomics into Nutrition Science. Addressing Epistemological and Social Challenges.* Valorisation Report. Nijmegen: Centre for Society and the Life Sciences (CSG), with Top Institute Food and Nutrition and the Pilot Plant.

5 The National Ecological Observatory Network serves as an exemplar of similar shifts in collaborative arrangements in terrestrial ecology.

6 Adams, JD, Black, GC, Clemmons, JR & Stephan, PE. (2005). “Scientific teams and institutional collaborations: Evidence from U.S. universities, 1981–1999”. *Research Policy*, 34(3): 259–285. p. 260.

7 Wutchy, S, Jones, BF & Uzzi, B. (2007). “The increasing dominance of teams in production of knowledge”. *Science*, 316: 1036–1039, pp. 1037.

8 Ibid. Supplementary Online Materials.

9 Adams et al., pp. 272–273.

10 Mattsson, P., Laget, P., Nilsson, A. & C. J. Sundberg (2008). “Intra-EU vs. extra-EU scientific co-publication patterns in EU”. *Scientometrics*, 75 (3): 555–574, p 572.

11 Ibid, p. 565.

12 Adams, J.D., Black, G. C., Clemmons J. R. & P. E. Stephan (2004) “Scientific teams and institution collaborations: evidence from U.S. Universities, 1981–1999”. *NBER Working Paper* 10640.

13 Hackett (2005) Hackett, E.J., (2005). “Introduction to the Special Guest-Edited Issue on Scientific Collaboration”. *Social Studies of Science*, 35(5), 667–672. op. cit note 10, p. 668.

14 Griesemer, J. and Gerson, E. (1993). “Collaboration in the Museum of Vertebrate Zoology”. Journal of the History of Biology 26(2): 185–203, p. 185.

15 For instance, computer gamers recently collaborated with scientists to uncover the structure of a protein important for the development of antiretroviral drugs for AIDS. See Faris et al. (2011). “Crystal structure of a monomeric retroviral protease solved by protein folding game”. *Naturel Structural and Molecular Biology* 18, 1175–1177.

16 Griesemer, J. & Gerson, E. p. 202.

17 Hackett (2005), op. cit. note 12, p. 671.

18 Shrum, W., Genuth, J., & Chompalov, I. (2007). *Structures of scientific collaboration*. Cambridge, MA: MIT Press.

19 Knorr-Cetina, K. (1999). *Epistemic cultures: how the sciences make knowledge*. Cambridge, MA: Harvard University Press. She presents molecular biology, as an 1individual, bodily lab-bench science’ with an individual ontology, although at the same time she notices an increased need for collaboration in biology, as knowledge and instruments become more complex and the field more competitive towards the end of her fieldwork in the lab which took mostly place in the 1980s.

20 Cook-Deegan, R. M. (1994). *The gene wars: science, politics, and the human genome*. New York, NY: Norton; Glasner, P. (1996). “From community to ‘collaboratory’? The Human Genome Mapping Project and the changing culture of science”. *Science and public policy*, 23(2), 109–116; Hilgartner, S. (1995). “The Human Genome Project”. In S. Jasanoff (Ed.), *Handbook of science and technology studies* (pp. 302–315). Thousand Oaks, CA: Sage; Sloan, P. R. (2000). *Controlling our destinies: historical, philosophical, ethical, and theological perspectives on the Human Genome Project*. Notre Dame, IN: University of Notre Dame Press; Venter, J. C. (2007). *A Life Decoded. My Genome: My Life*. New York, NY: Viking.

21 Roberts, L. (2001). “Controversial from the start”. *Science*, 291(5507):1182–1188. p. 1183.

22 Cf. Cummins, J. N., & Kiesler, S. (2005). “Collaborative research across disciplinary and organisational boundaries”. *Social Studies of Science*, 35(5), 703–722. Katz, J. S., & Martin, B. R. (1997). “What is research collaboration?” *Research Policy*, 26, 1–18.

23 Lemma “Systema Naturae.” Encyclopædia Britannica. 2008. Encyclopædia Britannica Online. Retrieved 25 August 2008 from http://www.britannica.com/EBchecked/topic/579163/-Systema-Naturae. See also Darwin, C. (1859). *On the Origin of Species by Means of Natural Selection, or the Preservation of Favoured Races in the Struggle for Life*. London: John Murray; Lamarck, J. B. (1809). *Zoological Philosophy: An Exposition with Regard to the Natural History of Animals* (H. Elliot, Trans.). Chicago: University of Chicago Press.

24 Blunt, W. (2001). The Compleat Naturalist: A Life of Linnaeus. London: Frances Lincoln.

25 Cart, T. W. (2004). *The Federal Fisheries Service, 1871–1940: Its Origins*, *Organisation, and Accomplishments.* Marine Fisheries Review, 66(4), pp. 1–46. Cf. International Polar Year. 2005. History of IPY. Available at: http://classic.ipy.org/development/history.htm [accessed February 14, 2007].

26 Dunbar, M. J. (1971). Anatomy of an Expedition. Geographical Review, 61(1), 161–163. p. 162.

27 Kwa, C. (1987), Representations of Nature Mediating between Ecology and Science Policy: the Case of the International Biological Programme. Social Studies of Science 17, 413–442.

28 Mcmasters, K. (2006). The Nuclear Neighbourhood. The New York Times, Nov 12.

29 Cf. Pickstone, J.V. (2000). *Ways of Knowing. A New History of Science, Technology and Medicine*. Manchester: Manchester University Press.

30 Geison, G.L. (1978). Micheal Foster and the Cambridge School of Physiology: The Scientific Enterprise in late Victorian Society. Princeton: Princeton University Press; Geison, G. L. (1993). “Research Schools and New Directions in the Historiography of Science”, in Geison, G. L. & F. L. Holmes (eds). Research Schools. Osiris, 8, 226–238.

31 Kay, L. E. (2000). Who wrote the book of life?: a history of the genetic code. Stanford: Stanford University Press.

32 Rasmussen, N. (2002). “Of ‘small men’, big science and bigger business: the second world war and biomedical research in the United States”. *Minerva, 40*, 115–146, p. 116.

33 Nature Editorial (2001). Post-genomic cultures. Nature, 409 (6822), 545.

34 Cases from ecological and environmental sciences include the Long-term Ecological Research Network (LTER), the National Centre for Ecological Analysis and Synthesis (NCEAS), the Resilience Alliance (RA), the Census of Marine Life (CoML), Decision Centre for a Desert City (DCDC) and Contaminated Land and Groundwater Megasites (SAFIRA II). Cases in molecular biology include the VIRology GenOmics consortium (VIRGO), the European Nutrigenomics Organisation (NUGO), the Gut Health project (IOPGH), the Top Institute Food and Nutrition (TIFN), the Human Genome Project (HGP), the Rice Genome Project (RGP) and the Silicon Cell Initiative (SiC). We personally conducted ten of these studies and we are familiar with others by existing analysis and literature. For the HGP, also see notes 2 and 20.

35 Indeed, Chunglin Kwa has argued that recent ecology and environmental science has been dominated by a ‘data bias’ driven by technology but which results in a fetishism of empiricism over theory and analysis. See e.g. Kwa, C. (2010) “A data bias in interdisciplinary cooperation in the sciences: Ecology and climate change,” Pages 161–178 in Collaboration in the New Life Sciences, Parker, Penders and Vermeulen, (eds).

36 See for more information on this consortium http://www.sysmo-db.org/consortium

37 See e.g. Kohler, R.E. (2002). Landcapes and Labscapes: Exploring the Lab–Field Border in Biology. University of Chicago Press and Garland E. (1975) Life Science in the Twentieth Century. New York: Wiley.

38 Kohler, (2002), p. 4.

39 See for more information http://www.biodiversity-exploratories.de and http://www.neoninc.org.

40 A point recently made in Parker, J.N.,Vermeulen, N. & Penders, B. (2011). Admin burden is part of the job. Nature 476: 33.

41 Sulston, J. and Ferry, G. (2002), p. 279.

42 Ibid, p. 209.

43 See e.g. Sidlauskas et al. (2009). Linking Big: The Continuing Promise of Evolutionary Synthesis. Evolution 64(4): 871–880, and Hampton, S.E & Parker, J.N. (2011). Collaboration and Productivity in Scientific Synthesis. *BioScience* 61(11): 900–910.

